# Genome-Wide Identification and Expression Analysis of the Thaumatin-like Protein Genes in *Filipendula ulmaria* under *Bipolaris sorokiniana* Infection

**DOI:** 10.3390/cimb48060640

**Published:** 2026-06-20

**Authors:** Ekaterina A. Istomina, Marina P. Slezina, Tatyana I. Odintsova

**Affiliations:** Vavilov Institute of General Genetics, Russian Academy of Sciences, 119333 Moscow, Russia; mer06@yandex.ru (E.A.I.); omey@list.ru (M.P.S.)

**Keywords:** plant immunity, thaumatin-like proteins (TLPs), *Filipendula ulmaria*, biotic stress, *Bipolaris sorokiniana*

## Abstract

Pathogenesis-related (PR) proteins are crucial for plant defense against pathogen infection. However, the specific role of thaumatin-like proteins (TLPs), which comprise the PR-5 family, in plant immune responses has not been thoroughly investigated. *Filipendula ulmaria* is a medicinal plant with valuable pharmacological properties, including antimicrobial, anti-inflammatory, gastroprotective, immunomodulatory, and anticancer activities. The structure of the TLP family and its role in the immune system of meadowsweet have not been studied so far. The goal of this study was to analyze in detail the TLP gene family in meadowsweet and explore its response to fungal infection. In the meadowsweet genome, we identified 27 putative TLP genes, examined their structure and location on chromosomes, analyzed *cis*-regulatory elements in the promoter regions, predicted the structure and physicochemical characteristics of the encoded proteins, and performed a phylogenetic analysis. We also studied the differential expression of TLP genes under *Bipolaris sorokiniana* infection. Of six differentially expressed genes, three genes were up-regulated 48 h post-infection, suggesting their involvement in defense response to the fungus. The results obtained shed light on the role of the TLP gene family in the immune system of *F. ulmaria* and form the foundation for the creation of disease-resistant crops in agriculture and the development of bio-based antimicrobials in medicine.

## 1. Introduction

Pathogen infection is one of the main causes of reduced crop yields, which are the primary source of food for the world’s population. An assessment of global agricultural production indicates that the total crop losses caused by pathogen infection amount to 20–40% [1]. The use of chemical pesticides and the development of resistant varieties are the main strategies for protecting plants from diseases. Currently, pesticides play a key role in maintaining crop yields and ensuring food security, as without their use, yield losses could reach up to 78% for fruits, 54% for vegetables, and 32% for grains [2]. However, pesticides can be toxic not only to pathogens but to other organisms as well, including birds, fish, beneficial insects, and non-target plants. They also pollute air, water, soil, and agricultural crops, leading to environmental pollution in general and contamination of food products in particular [2]. All these factors negatively impact human health, causing various diseases, from acute poisoning to cancer. Additionally, climate change also influences pesticide use, contributing to increased application and, consequently, pollution. Besides the negative effects on ecology and human health, the use of pesticides boosts the development of antimicrobial resistance in pathogenic microorganisms.

In the context of sustainable agriculture, there is a need for effective and safe biopesticides that eliminate pests but pose minimal risks to the environment. In recent years, proteins and peptides have become a promising area in plant protection due to their high inhibitory activity and ecological compatibility. Of prime interest are defense proteins of the plant itself.

Throughout their lives, plants encounter diverse stressful conditions. To withstand environmental challenges, they have developed physical and chemical defense mechanisms, one of which is the production of pathogenesis-related proteins (PR proteins). PR proteins play a pivotal role in plant defense against biotic and abiotic stressors. They were first discovered in tobacco plants with a hypersensitive response to tobacco mosaic virus infection. Accumulation of PR proteins was closely associated with the intensity of symptoms observed on the infected plants [3,4]. In subsequent studies on proteins induced in response to pathogens, PR proteins were discovered in different plant species. Diverse in structure and mode of action, PR proteins have been categorized into 19 classes [5]. Most of them possess antimicrobial properties in vitro. Transgenic plants expressing PR protein genes display enhanced resistance to pathogens [6].

The PR-5 protein family is represented by related polypeptides with sequence similarity to thaumatin, a sweet-tasting protein from the fruits of the shrub *Thaumatococcus danielli* found in West Africa, and therefore referred to as thaumatin-like proteins (TLPs) [7,8,9]. This protein family contains acidic, neutral, and very basic members, which are located extracellularly or within vacuoles. Most PR-5 proteins share a highly conserved thaumatin family signature G-x-{GF}-x-C-x-T-{GA}-D-C-x(1,2)-{GQ}-x(2,3)-C [8]. Two types of TLPs have been described in plants: large TLPs (L-type TLPs) with a molecular weight ranging from 20 to 26 kDa and containing 16 conserved cysteine residues forming 8 intra-chain disulfide bonds, and small TLPs (S-type TLPs) with a molecular weight of approximately 17 kDa, and only 10 conserved cysteine residues forming five disulfide bonds, which are found in monocots and conifers [7]. TLPs possess a highly conserved three-dimensional structure composed of three domains and a cleft between domains I and II [10]. This cleft can exhibit acidic, neutral, or basic properties, enabling it to bind various ligands or receptors. The acidic cleft composed of highly conserved amino acids REDDD is particularly associated with antifungal activity [7].

The first report on the biological activity of PR-5 proteins was the discovery that zeamatin, a 22-kDa thaumatin-like protein from maize (*Zea mays*), was antifungal and displayed membrane-permeabilizing activity [11]. It was then shown that tobacco osmotin and its tomato analog were active against *Phytophtlora infestans* [12]. The results demonstrating inhibition of fungal hyphal growth or spore germination by PR-5 proteins were extended to different plant species by in vitro antifungal assays. However, there are no reports on antibacterial activity for these proteins. Comparative studies of the activity spectrum of individual family proteins reveal significant differences [13]. Thus, each protein likely exhibits characteristic specificity toward certain fungi depending on its amino acid sequence. In an early study, two basic TLPs, R and S, from barley (*Hordeum vulgare*) grain, were shown to inhibit growth of *Trichoderma viride* and *Candida albicans,* acting synergistically with barley grain chitinase C [14]. A recombinant thaumatin-like protein from naked oat (*Avena nuda*) exhibited antifungal activity against *Fusarium oxysporum* [15]. The protein also caused membrane permeabilization and accumulation of reactive oxygen species in the mycelium of *F. oxysporum*. Three TLPs from leaves of barley infected with *Rhynchosporium secalis* exhibited antifungal activity [16]. A TLP with antifungal activity against *Candida* species, whose expression was induced by laminarin oligosaccharides and salicylic acid, was isolated from *Cassia didymobotrya* cell cultures [17]. BanTLP from banana significantly suppressed the growth of *Penicillium expansum* and caused morphological changes in the fungus. It was demonstrated that BanTLP affected the fungal cell wall, disrupting its integrity and increasing permeability, leading to fungal cell death [18]. However, it should be noted that some PR-5-like proteins, including thaumatin itself, an acidic PR-5-like protein from cherry fruits, and certain pathogen-induced acidic PR-5-like proteins of tobacco, apparently do not possess antifungal activity [13].

The mode of action of PR-5 proteins is not completely understood. It has been suggested that the antifungal properties of TLPs are associated with hydrolysis of fungal cell wall glucans or inhibition of cell-wall degrading enzymes (e.g., fungal xylanase) [19,20,21], as well as with the ability to destroy fungal cell membranes and induce programmed cell death [7]. Indeed, some TLPs exhibit endo-β-1,3-glucanase activity [22,23]. However, it remains unclear whether glucan binding and glucanase activity are necessary for the antifungal activity of PR-5 proteins. For example, two TLPs from germinated barley PR-5b and HvPR5c differed significantly in binding insoluble (1,3)-β-D-glucan, and neither displayed glucanase activity. However, both inhibited fungal growth in vitro [24].

Besides in vitro tests of isolated proteins, the defensive role of PR-5 proteins has been confirmed by induction of gene expression by biotic and abiotic stressful factors and enhanced tolerance to stresses of transgenic plants overexpressing PR-5 protein genes [25,26,27,28].

Studies employing genome-wide analysis of a range of plant species, such as barley, tomato, cotton, garlic, poplar, bamboo, grape, etc., demonstrated that thaumatin-like proteins are the products of a large and complex gene family involved in host defense and developmental processes [29,30,31,32,33,34,35,36,37,38]. However, the roles of individual members of the TLP superfamily remain largely unknown.

*Filipendula ulmaria* (Rosaceae family), or meadowsweet, is a perennial herbaceous plant that grows on wet ground in swamps, marshes, wet woods, meadows, and by rivers. It is a medicinal plant with valuable pharmacological properties. Extracts from this plant exhibit anti-inflammatory, antimicrobial, gastroprotective, immunomodulatory, and anticancer activities [39]. The advantageous pharmacological characteristics of meadowsweet have historically been ascribed to its secondary metabolites. However, recent research indicates that the antimicrobial effects of *F. ulmaria* are at least partially due to the presence of defense peptides (AMPs) in this remarkable herb [40]. In this work, we extend our search for polypeptide-based antimicrobial compounds in meadowsweet and focus on the enigmatic PR-5 protein family.

The aim of this study was to perform genomic and transcriptomic analyses of the TLP gene family in *F. ulmaria* to broaden our understanding of these proteins, which are an integral part of the plant’s defense system, and pave the way for innovative strategies of crop protection. In this work, we studied the structure of meadowsweet genes and their location on chromosomes, predicted the structure and physicochemical characteristics of the encoded proteins, their domain structure, and subcellular localization. We analyzed the *cis*-acting regulatory elements in the promoter regions of *F. ulmaria* TLP genes and explored their differential expression in response to infection with the pathogenic fungus *Bipolaris sorokiniana* to shed light on the role of the TLPs in stress response. Harnessing the potential of meadowsweet TLPs holds promise for various agricultural and biomedical applications, including the development of resistant crops, bio-based products, and pharmaceuticals.

## 2. Materials and Methods

### 2.1. Search for TLP Genes in the Genomic Data of F. ulmaria

The search for TLP genes was conducted using the genome assembly of *F. ulmaria*, drFilUlma1 (Accession PRJEB77879), which contained nucleotide sequences of all seven chromosomes. As a query for identifying TLPs from the Rosaceae family, motifs of thaumatin-like proteins (cd09218, pfam00314) from the NCBI CDD database (https://www.ncbi.nlm.nih.gov/cdd/, accessed on 23 December 2025) were used with the InterProScan program [41,42]. Then *F. ulmaria* genome was aligned with the TLP protein sequences from the Rosaceae family using the NCBI TBLASTN search algorithm (https://blast.ncbi.nlm.nih.gov/Blast.cgi, accessed on 15 January 2026) with an E-value cut-off of <1 × 10^−10^ [43]. Open reading frames were analyzed with the Augustus software (http://bioinf.uni-greifswald.de/augustus/submission.php, accessed on 15 January 2026) and FGENESH (http://www.softberry.com/berry.phtml?topic=fgenesh&group=help&subgroup=gfind, accessed on 15 January 2026) [44,45]. To identify conserved domains within the proteins, InterProScan was employed (https://www.ebi.ac.uk/interpro/search/sequence/, accessed on 19 February 2026) [42], and only the sequences containing the pfam00314 domain were considered to be the TLP sequences.

Nucleotide and protein sequences of the identified members of the *FuTLP* gene family were compared with the complete genome sequences of *F. ulmaria* using BLAST (https://blast.ncbi.nlm.nih.gov/Blast.cgi, accesed on 21 January 2026), and the corresponding genomic sequences, chromosomal positions, and patterns of exon–intron distribution for each family member were obtained. To localize thaumatin genes on chromosomes, the online program MF2C v2.1 (http://mg2c.iask.in/mg2c_v2.1, accessed on 4 February 2026) was used [46]. For visualization of exon–intron structures of the genes, the online program Gene Structure Display Server 2.0 (https://gsds.gao-lab.org/Gsds_about.php, accessed on 5 February 2026) was employed [47].

### 2.2. Experimental Design

Seeds of *Filipendula ulmaria* (L.) Maxim. used in the experiment were collected in the Moscow region (Russia). *Bipolaris sorokiniana* strain VKM F-4006 was sourced from the State Collection of Plant Pathogenic Microorganisms at the All-Russian Research Institute of Phytopathology (Moscow region, Russia). The fungus was cultured as described earlier [48]. Seeds of *F. ulmaria* were surface-sterilized by soaking in 70% ethanol for 5 min, followed by three rinses with a large volume of sterile water. The sterilized seeds were sown in a mixture of soil and perlite (ratio 1:2, *v*/*v*), which had been pre-steamed, into four plastic containers containing 100 seeds each. To induce cold stratification, the sealed containers were kept in the dark at +8 °C for three months. Afterward, they were moved to a climate chamber set at +25 °C for two weeks until the seedlings developed two true leaves. At this point, plants from each container were divided into two groups: one inoculated with the pathogen (*B. sorokiniana*) and the other serving as a mock-inoculated control, with each group transplanted into separate new containers. This process resulted in four experimental replicates. For inoculation, a freshly prepared conidial suspension (7 mL per container, at a concentration of 0.5 × 10^6^ conidia/mL) was applied to the leaves. Control plants received the same volume of sterile water. After treatment, all plants were kept in the climate chamber at +25 °C with a 16 h light/8 h dark photoperiod. For RNA isolation, four samples of plant tissue from the control and infected groups were taken 24 and 48 h post-inoculation (hpi) or water treatment. These samples were labeled as control 1 and 3 (at 24 and 48 hpi, respectively) and infected 2 and 4 (at 24 and 48 hpi, respectively). For each experimental group, a pooled sample consisting of 8–12 whole plants (200–250 mg of plant tissue) from four containers was cut into pieces and split into two equal parts (replicates a and b), each preserved in 1 mL of IntactRNA reagent (Eurogen, Moscow, Russia) within Eppendorf tubes.

### 2.3. RNA Isolation, Library Preparation and Next-Generation Sequencing (NGS)

Total RNA was extracted using the Plant RNA Isolation Aid kit (Ambion, ThermoFisher, Waltham, MA, USA) following the manufacturer’s instructions. The RNA concentration was measured with a Qubit fluorimeter (Invitrogen, ThermoFisher, Waltham, MA, USA), and the RNA quality was assessed using an Agilent 2100 Bioanalyzer (Agilent Technologies, Santa Clara, CA, USA). Half of each RNA sample was used to prepare eight cDNA libraries for sequencing, while the other half was reserved for validation via qRT-PCR.

After enrichment of mRNA using oligo(dT) beads, cDNA libraries (1a, 1b, 2a, 2b, 3a, 3b, 4a, and 4b) were constructed using the TruSeq Stranded mRNA Library Prep Kit (Illumina) according to the manufacturer’s protocol. The quality of these libraries was checked with an Agilent 2100 Bioanalyzer. Sequencing was performed on the Illumina NextSeq 500 platform (Illumina, San Diego, CA, USA) at the EIMB RAS “Genome” Center. For libraries 1a, 1b, 2a, and 2b, 75 bp single-end reads were generated, while for libraries 3a, 3b, 4a, and 4b, 150 bp paired-end reads were obtained. The resulting FASTQ files were generated using the bcl2fastq Conversion Software v1.8.4 (Illumina).

### 2.4. Search for TLP Genes in the Transcriptomic Data of F. ulmaria

Sequencing data were deposited in NCBI at BioProject accession number PRJNA1047088 [48]. Transcript assembly was performed using the rnaSPAdes v3.15.4 program [49]. TransDecoder v5.5.0 was used to identify open reading frames in contigs [50].

Differential gene expression analysis was based on read counts in infected plants compared to uninfected controls. Differentially expressed genes (DEGs) were identified using the DESeq2 package [51]. Clean reads were aligned to the combined transcriptome assembly using the BWA MEM algorithm and SAMtools [52,53]. Expression levels were calculated as counts per million (CPM) reads. Genes with |log2FoldChange| > 1 and *p* < 0.05 were considered DEGs.

### 2.5. qRT-PCR Analysis

To validate the transcriptomic data, 12 *trFuTLP* genes were analyzed using PCR with the specific primers flanking the open reading frame region (Table S1). Another set of primers was used for validation of gene expression levels by real-time PCR (Table S2). Primers were selected using the Primer Blast program (https://www.ncbi.nlm.nih.gov/tools/primer-blast/index.cgi, accessed on 26 February 2026). The optimal annealing temperature for each set of primers on the cDNA template was determined by gradient PCR, varying the temperature in the range from 55 °C to 65 °C. The specificity of amplification was assessed by analyzing the melting curves of PCR fragments, as well as by electrophoretic separation of PCR products in a 3% agarose gel. The *EF1-α* gene of *F. ulmaria* was used as an internal control. The obtained characteristics of the calibration curve (slope = −3.65, amplification efficiency (E) = 87.9%, determination coefficient (R2) = 0.99974) indicate high linearity and acceptable efficiency of the PCR reaction for the *EF1-α* gene. *F. ulmaria* RNA obtained by pooling RNA samples from two replicates of the same sample in equal proportions was used for cDNA synthesis with oligo(dT)-primers and the Mint cDNA synthesis kit (Evrogen, Moscow, Russia) following the manufacturer’s instructions.

Quantitative RT-PCR was performed using the qPCRmix-HS SYBR+HighROX kit (Evrogen) according to the manufacturer’s protocol on a DT-96 Real-Time instrument (DNA-technology, Moscow, Russia). PCR conditions were as follows: initial denaturation at 94 °C for 2 min; 40 cycles of denaturation at 94 °C for 30 s; primer annealing at 58–60 °C for 30 s; primer extension at 72 °C for 30 s; with a final extension at 72 °C for 5 min. Each experiment was performed in three technical replicates. The specificity of PCR amplification was confirmed by sequencing the PCR product. Relative transcript levels were assessed using the 2^−ΔΔCT^ method [54]. Results were presented as mean ± standard error (SE).

### 2.6. Characterization of the TLPs

For annotation and homology identification between TLPs discovered in the genome and transcriptomes (FuTLPs and trFuTLPs), the BLAST program and the GenBank database were used [43,55]. The presence of signal sequences and transmembrane helices (TMHs) in proteins was predicted using the server SignalP 6.0 (https://services.healthtech.dtu.dk/services/SignalP-6.0/, accessed on 17 February 2026) and TMHMM - 2.0 (https://services.healthtech.dtu.dk/services/TMHMM-2.0/, accessed on 17 February 2026), respectively [56]. Physicochemical properties of TLPs were predicted using the ProtParam program (http://web.expasy.org/protparam/, accessed on 2 March 2026) [57]. Net charge at pH 7, pI, GRAVY index, and aliphatic index were computed for protein sequences without signal peptides. Subcellular localization was determined using the program Cell-PLoc 2.0 (http://www.csbio.sjtu.edu.cn/bioinf/Cell-PLoc-2/, accessed on 2 March 2026) [58]. Conserved motifs in TLPs, with a maximum of 7 motifs and an optimal motif width of 20–50 residues, were identified using the MEME program (https://meme-suite.org/meme/tools/meme, accessed on 20 May 2026) [59]. *Cis*-acting regulatory elements in the promoter region 2000 bp upstream of each TLP gene’s start codon were predicted with the PlantCARE database (https://bioinformatics.psb.ugent.be/webtools/plantcare/html/, accessed on 12 May 2026) [60].

Sequence alignment was performed using the Vector NTI Advance 9 software (Invitrogen, Waltham, MA, USA). The phylogenetic tree of the TLPs was constructed using the Maximum Likelihood method in MEGA X [61], with a bootstrap value of 1000. The three-dimensional structures of the identified meadowsweet TLPs were predicted using SWISS-MODEL (https://swissmodel.expasy.org/interactive, accessed on 10 March 2026) [62]. In addition, the quality of the predicted protein structures was evaluated using VERIFY 3D (https://saves.mbi.ucla.edu/, accessed on 12 March 2026) and ProQ server (https://proq.bioinfo.se/cgi-bin/ProQ/ProQ.cgi, accessed on 12 March 2026) [63,64].

## 3. Results

### 3.1. Search for Thaumatin-like Protein Genes in the Genome Data of F. ulmaria

**Figure 1:**
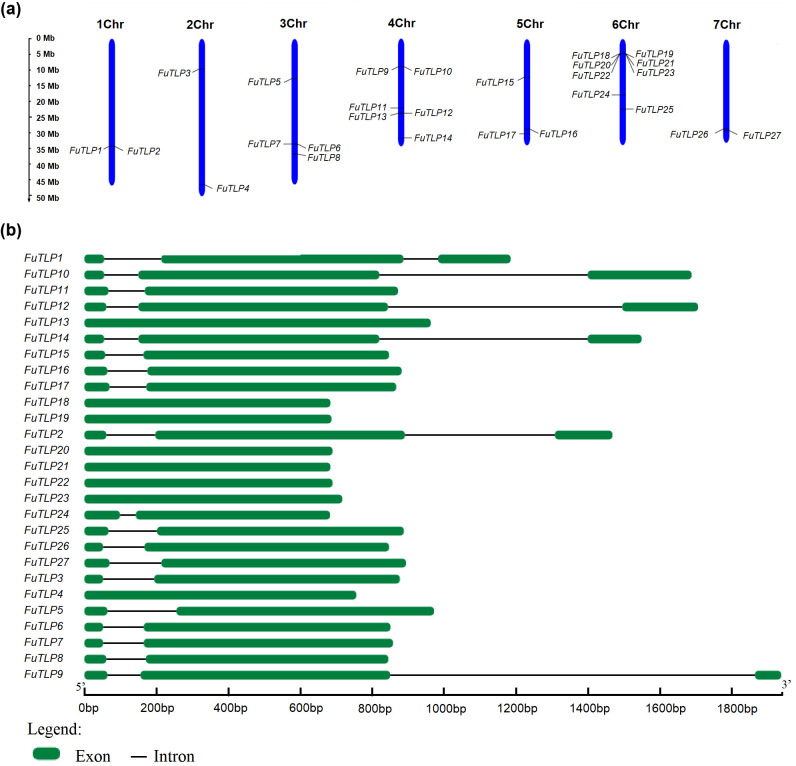
Analysis of *F. ulmaria* TLP genes: (**a**) Distribution on chromosomes; (**b**) exon–intron structure.

In the meadowsweet genome, 27 full-length genes encoding TLP precursors were identified. All detected genes were organized according to their relative linear positions on the chromosomes and named from *FuTLP1* to *FuTLP27* accordingly (Table S3, Figure 1a). The *FuTLP* genes were found on all seven chromosomes; however, their distribution on the chromosomes was uneven. For example, only two genes were identified on chromosomes 1, 2, and 7, while the highest number of genes (8) was found on chromosome 6 (Figure 1a).

Aligning the coding regions of the TLP genes with complete genomic nucleotide sequences revealed that 13 *FuTLP* genes contained one intron, and six genes had two introns (Figure 1b); eight genes carried no introns.

The characteristics of the translated proteins are shown in Table 1. All identified proteins showed homology with the TLP family of Rosaceae, with the percentage of homology ranging from 57 to 91%. BLAST annotation showed that 21 FuTLP sequences showed homology to thaumatin-like and osmotin-like proteins of the PR-5 family (Table 1). Two pairs of proteins (FuTLP14 and 24; FuTLP6 and 7) exhibited sequence similarity to hypothetical proteins from *Rubus argutus* and *Malus domestica*, respectively. Two proteins, FuTLP20 and FuTLP22, displayed sequence similarity to the P21 protein of *R. chinensis*.

The length of predicted FuTLP proteins ranged from 212 to 337 amino acid residues, and their molecular weights varied from 23.2 to 34.5 kDa (Table 1). All 27 FuTLP precursors were predicted to contain a signal peptide, with a length varying from 19 to 30 amino acid residues. Eight proteins were predicted to have one transmembrane domain, while FuTLP17 had two transmembrane domains (Table 1). It was established that six of the identified FuTLPs are found in vacuoles, while the others are localized extracellularly.

The pI values of the proteins ranged from 4.20 to 9.18: for 16 FuTLPs, pI values were in the acidic range from 4.20 to 6.77; three proteins had pI = 7.50 (FuTLP14), 7.52 (FuTLP5), and 7.54 (FuTLP8); and for eight proteins, pI was in the alkaline region from 7.92 to 9.18 (Table 1).

The aliphatic index of the identified FuTLPs varied from 41.74 to 78.44, characterizing these proteins as moderately thermostable (Table 1). The GRAVY index was in the range from −0.464 to 0.174, with 22 FuTLPs having negative GRAVY values. A negative GRAVY value indicates that the protein is hydrophilic and highly soluble in water.

### 3.2. Search for Thaumatin-like Protein Genes in Transcriptomic Data of Meadowsweet After Infection with B. sorokiniana

As a result of transcriptomic analysis of *F. ulmaria* infected with *B. sorokiniana*, 12 TLP genes were identified (Table S4); their sequences were confirmed by PCR. All sequences detected showed homology to *FuTLP* genes and were named *trFuTLP*s, and their numbers corresponded to those of their genomic counterparts. The results of the comparison of *FuTLP*s and *trFuTLP*s sequences are presented in Table 2. Three *trFuTLP*s (*trFuTLP14*, *21*, *23*) had 100% sequence similarity at the nucleotide and amino acid levels with their respective homologs found in the genome. *trFuTLP2* had only one nucleotide substitution and no amino acid substitutions compared to *FuTLP2*. The maximum number of nucleotide substitutions (32) was observed in *trFuTLP20*, resulting in 12 amino acid substitutions in the protein sequence compared to the corresponding FuTLP homolog, followed by *trFuTLP22* (20 nucleotide and 10 amino acid substitutions) and *trFuTLP18* (11 nucleotide and 7 amino acid substitutions) (Table 2). The occurrence of nucleotide and amino acid substitutions in transcriptomic data compared to genomic data might reflect the genetic diversity, including allelic variation, mutations, etc., of TLP genes within the species.

All identified trFuTLP proteins exhibited homology to the TLP family of Rosaceae plants, with homology levels ranging from 75% to 95% (Table 3). Among the homologs were thaumatin-like proteins of the PR-5 family of *Rosa rugosa*, *R. chinensis*, *R. sericea*, and *Fragaria vesca*, an osmotin-like protein of *R. rugosa* (trFuTLP4), a hypothetical protein from *Rubus argutus* (trFuTLP14), and protein P21 of *R. chinensis* (trFuTLP20 and trFuTLP22).

The length of trFuTLPs ranged from 227 to 337 amino acid residues (Table 3). For all trFuTLP precursors, the presence of a signal peptide was predicted, ranging in length from 19 to 30 amino acids. Four TLPs were predicted to contain transmembrane domains (Table 3). Seven thaumatin-like proteins are localized extracellularly, and five are in vacuoles.

The pI values varied from 4.29 to 8.48, with seven proteins having pI in the acidic range (Table 3). The aliphatic index of trFuTLPs ranged from 41.29 to 73.00, characterizing these proteins as moderately thermostable. The GRAVY index values ranged from −0.463 to 0.174, with nine trFuTLPs having negative GRAVY values, indicating hydrophilic properties, while the other three exhibited more hydrophobic properties.

### 3.3. Amino Acid Sequences of Thaumatin-like Proteins

**Figure 2:**
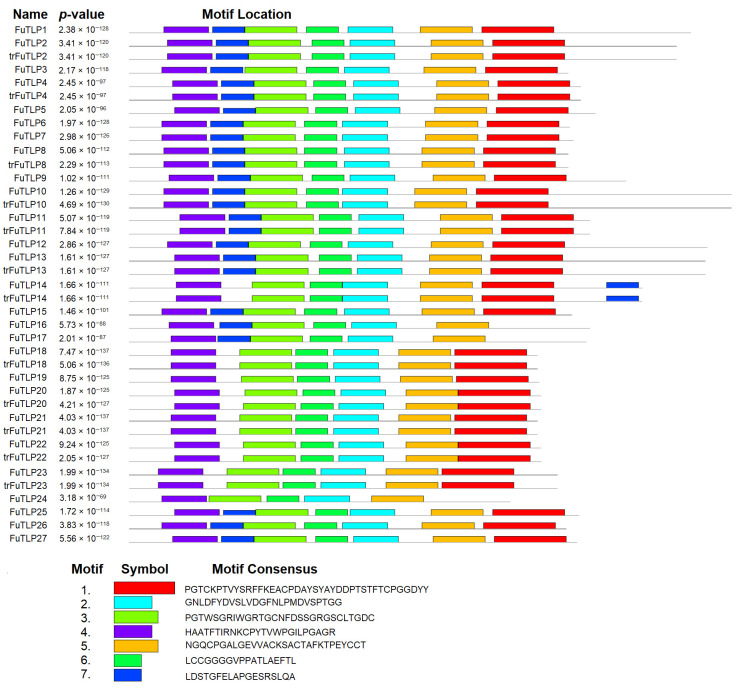
Distribution of conserved motifs in meadowsweet TLP sequences, predicted with the MEME program [59].

The amino acid sequences of TLPs from meadowsweet are shown in Figure S1. All the identified sequences contained a conserved motif characteristic of thaumatin-like proteins: G-x-{GF}-x-C-x-T-{GA}-D-C-x(1,2)-{GQ}-x(2,3)-C and 16 cysteine residues (Figure 2). FuTLP15 had two extra cysteine residues neighboring C2 and C8; FuTLP8 (and the corresponding homolog trFuTLP8) and FuTLP27 had an extra cysteine residue before C8 and C7, respectively; and FuTLP11 and trFuTLP11 had an extra residue between C14 and C15 (Figure S1). Three TLP sequences (FuTLP1, FuTLP2, and FuTLP10) and the corresponding homologs had one additional cysteine residue in the C-terminal prodomain, while FuTLP14 and trFuTLP14 had two cysteine residues in this domain. The REDDD motif is fully conserved in 33 of the 39 TLP sequences, except for FuTLP4, trFuTLP4, FuTLP5, FuTLP17, FuTLP19, and FuTLP24 (Figure S1).

The presence of conserved motifs in FuTLPs and trFuTLPs was predicted using the MEME program [59]. We identified seven conserved motifs in FuTLP and trFuTLP sequences. Their distribution is shown in Figure 2. In most proteins, the motifs were arranged in the order 4-7-3-6-2-5-1; however, some proteins (FuTLP18, 19, 20, 21, 22, 23, 24, and the corresponding homologs) did not contain the 7th motif, and in FuTLP14 and trFuTLP14, the 7th motif is located at the very end of the molecule.

### 3.4. Modeling of the Three-Dimensional Structure of TLPs

**Figure 3:**
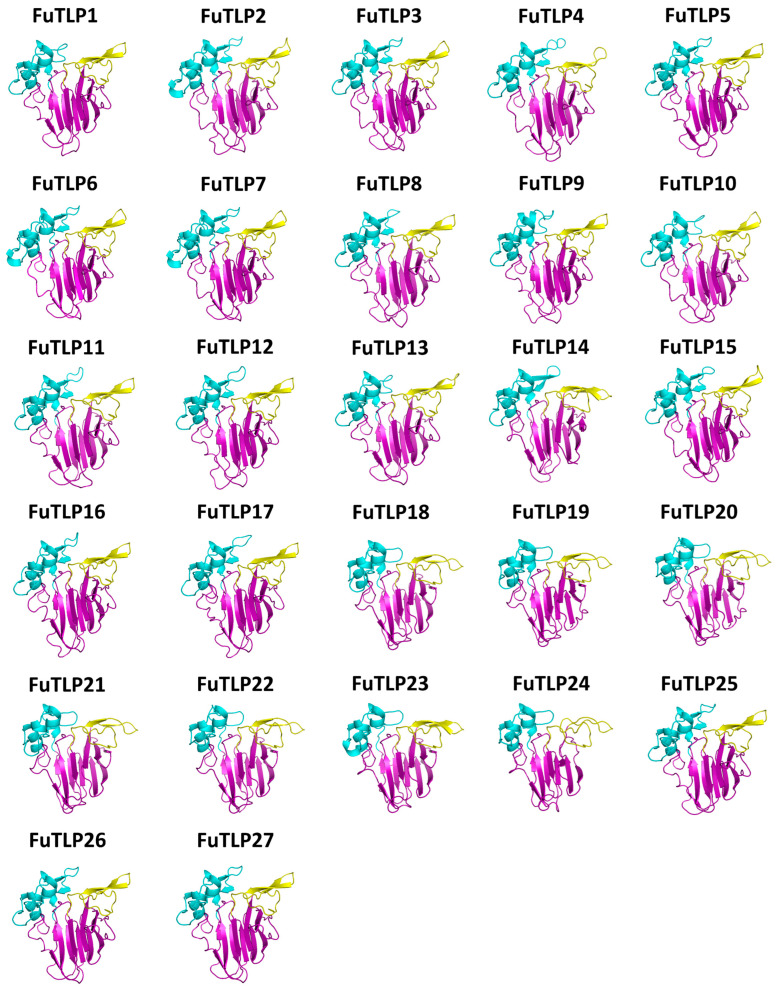
Predicted 3D structures of FuTLPs (cartoon representation). Modeling was performed using SWISS-MODEL [62]. Domains I, II, and III are shown in purple, cyan, and yellow, respectively.

The 3D structure of FuTLPs was predicted using the homology-modeling server SWISS-MODEL (Figure 3) [62]. The best templates for the meadowsweet TLPs were 1Z3Q (Mus a 4, *Musa acuminata*), 3ZS3 (Mal d 2, *Malus domestica*), 2AHN (Pru av 2, *Prunus avium*), 4L2J (Osmotin, *Calotropis procera*), and 7P20 (Jun a 3, *Juniperus ashei*), whose crystallographic structures have been determined. The identity of FuTLPs and templates ranged from 42.73 to 83.00% (Table S5). The assessment of the quality of the models obtained showed that the predicted 3D structures of FuTLPs were quite reliable (Table S5).

Thaumatin-like proteins from meadowsweet, like typical TLPs, have a three-dimensional structure with three conserved domains, namely I, II, and III (Figure 3). Domain I is a lectin-like β-barrel consisting of 9–11 β-strands, while domain II is composed of 4–5 α-helical regions and loops (domain II of several TLPs also contains two antiparallel β-strands); domain III consists of a small loop and two antiparallel β-strands, with the exception of FuTLP24, in which domain III is represented by an unstructured coil (Figure 3). Two domains, I and II, form a central V-shaped cleft on the surface of the molecule, which contains predominantly hydrophilic residues (Figure 3 and Figure S2). In almost all meadowsweet TLPs, the cleft is acidic due to the presence of five highly conserved amino acid residues (arginine, glutamic acid, and three aspartic acid residues) (Figure S2), which are supposed to provide the antifungal function of TLPs [7]. The exception is FuTLP24, whose cleft is neutral because of three amino acid substitutions (Figures S1 and S2).

### 3.5. Phylogenetic Analysis of TLPs

**Figure 4:**
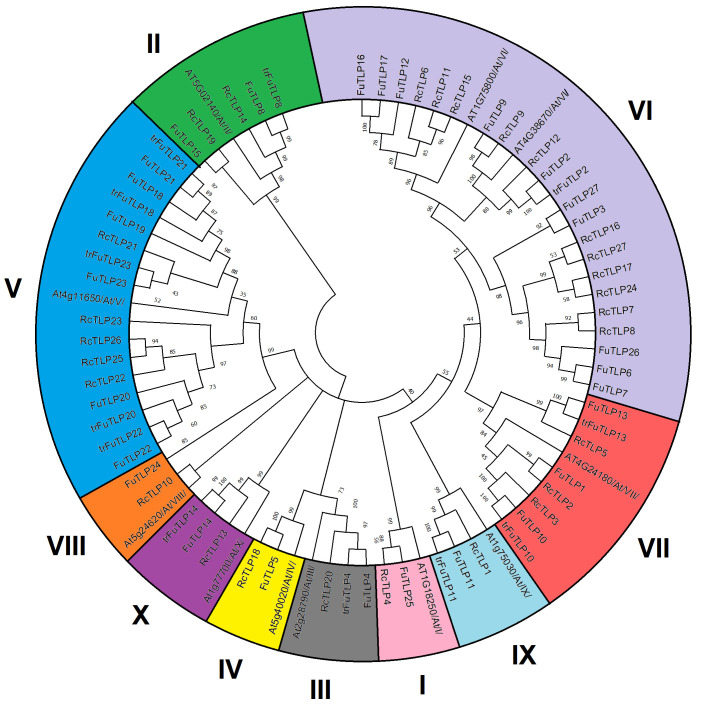
A phylogenetic tree of *F. ulmaria* TLPs and selected TLPs of *A. thaliana* (At) and *R. chinensis* (Rc) [36,65]. The tree was constructed with MEGA X software using the Maximum Likelihood method; bootstrapping was performed 1000 times to obtain support values for each branch [61]. All identified *F. ulmaria* TLPs were clustered into 10 groups (I to X), shown by different colors.

In the phylogenetic analysis of TLPs from meadowsweet, 11 TLP sequences from *A. thaliana* and 27 RcTLP sequences from *Rosa chinensis* were used as references. The distribution of meadowsweet TLPs was uneven. Thirty-nine FuTLPs (27 FuTLPs and 12 trFuTLPs) fell into 10 phylogenetic groups labeled from I to X (Figure 4). The most numerous groups, V and VI, included 11 TLPs each: Group V comprised FuTLP18, 19, 20, 21, 22, and 23, and the corresponding homologs trFuTlp18, 20, 21, 22, and 23; Group VI—FuTLP2, 3, 6, 7, 9, 12, 16, 17, 26, 27, and trFuTLP2 (Figure 4). Group VII contained five TLPs: FuTLP1, 10, 13, and trFuTLP10 and 13. Group II included three TLPs: FuTLP8, trFuTLP8, and FuTLP15. Some groups comprised one TLP along with its transcriptomic homolog (Group III, FuTLP 4 and trFuTLP4; Group IX, FuTLP11 and trFuTLP11; Group X, FuTLP14 and trFuTLP14). Group I contained a single protein, FuTLP25, as well as Group IV (FuTLP5) and Group VIII (FuTLP24).

### 3.6. Analysis of Differentially Expressed TLP Genes upon Infection with B. sorokiniana

**Figure 5:**
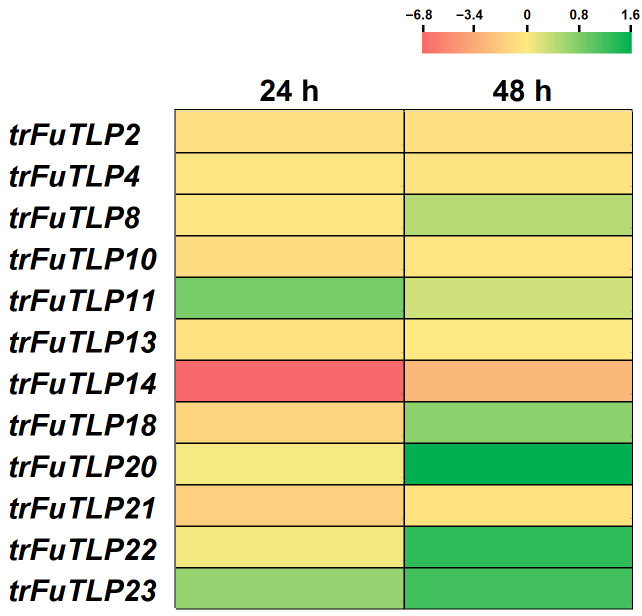
Expression profiles of *trFuTLP* genes at 24 and 48 h after infection with *B. sorokiniana*. The color gradient indicates expression changes from low (red) to high (green).

Transcriptomic analysis of *F. ulmaria* after infection with *B. sorokiniana* at 24 and 48 h post-infection revealed 12 *trFuTLP* genes (Figure 5). Transcriptional profiling showed that the expression of 6 out of 12 genes changed significantly in response to infection (|log2FoldChange| > 1) (Figure 5, Table S6). At 24 h after pathogen inoculation, the expression of three genes, *trFuTLP14*, *trFuTLP18,* and *trFuTLP21*, decreased relative to the control; moreover, the expression of the *trFuTLP14* gene also decreased 48 h after infection. After 48 h, the expression of three other genes, *trFuTLP20*, *trFuTLP22*, and *trFuTLP23*, increased, with log2FoldChange values ranging from 1.11 to 1.60 (Figure 5). The remaining six genes did not show differential expression. Notably, only the genes from one phylogenetic group V were up-regulated in meadowsweet after infection with the fungus.

**Figure 6:**
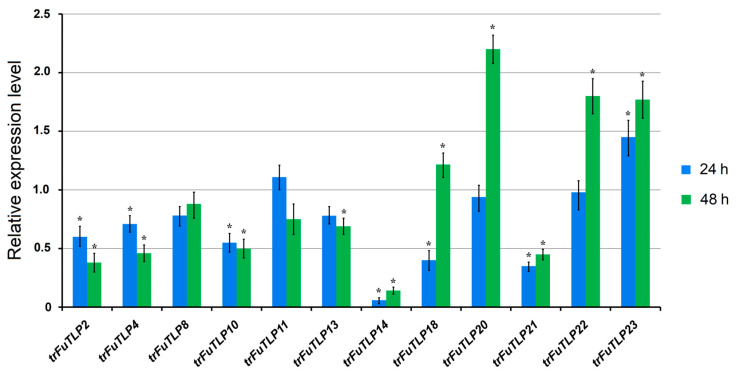
qRT-PCR validation of expression levels for *trFuTLP* genes in response to *B. sorokiniana* infection at 24 and 48 h post-infection. Relative expression values were normalized using the *EF1-α* gene as an internal control and standardized relative to the control values. Bars represent mean ± standard error (SE). Asterisks indicate significant differences between infected and control plants (Student’s *t*-test, *p* < 0.05; *n* = 3).

To confirm the expression levels of *trFuTLP* genes, quantitative real-time PCR was performed with all *trFuTLP* genes after infection with *B. sorokiniana* at 24 and 48 h (Figure 6). Increased expression of the *trFuTLP23* gene was observed at 24 h, and of *trFuTLP20*, *22*, and *23* at 48 h. Decreased expression levels were observed for the *trFuTLP2*, *4*, *10*, *14*, *18*, and *21* genes at 24 h, and at 48 h for *trFuTLP2*, *4*, *10*, *14*, and *21*. Accordingly, the results of real-time PCR matched the transcriptomic data, although the expression levels at 48 h were slightly lower than expected.

### 3.7. Cis-Acting Regulatory Elements in the Promoter Regions of FuTLP Genes

**Figure 7:**
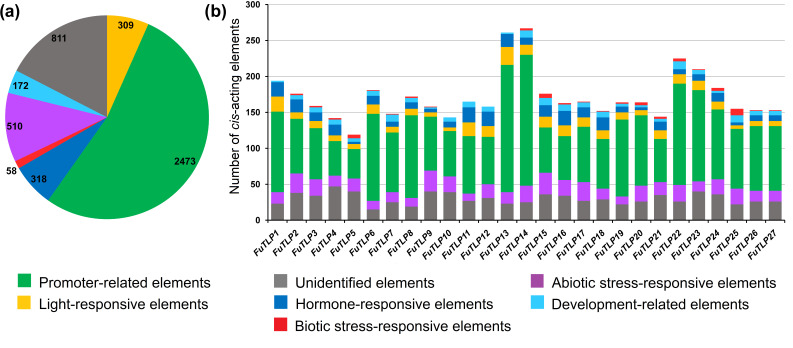
Statistics for predicted *cis*-acting regulatory elements in the promoters of *FuTLP* genes: (**a**) Distribution of the total number of *cis*-acting elements across biological processes for all *FuTLP* genes; (**b**) representation of *cis*-acting elements of different categories in the promoters of *FuTLP* genes.

The *cis*-acting elements (CEs) in the gene promoter region are responsible for the regulation of gene expression, mainly by binding to specific transcription factors, during plant development and under different environmental conditions. To reveal potential CEs controlling *FuTLP* gene expression, the promoter sequence 2000 bp upstream of the start codon site was analyzed. A total of 93 different types of *cis*-acting regulatory elements were identified, with their total number reaching 4651. These elements were divided into seven groups depending on the biological processes they are putatively involved in: (1) promoter-related elements, (2) light-responsive elements, (3) elements responsive to abiotic stress, (4) elements responsive to biotic stress, (5) hormone-responsive elements, (6) development-related elements, and (7) unidentified *cis*-acting elements (Figure 7, Table S7).

The number of promoter-related CEs, specifically TATA-box, CAAT-box, AT~TATA-box, A-box, and TATA, totaled 2473; their number ranged from 60 in *FuTLP21* to 182 in *FuTLP14* (Figure 7, Table S7). The second largest group consisted of 811 CEs that were not functionally characterized. They were distributed from 15 in *FuTLP6* to 47 in *FuTLP4*. Another group of CEs, consisting of 309 sites, is responsive to light (Figure 7, Table S7). The most numerous CEs in this group are Box 4, G-box, GT1-motif, GATA-motif, and Sp1. The number of elements ranged from 5 in *FuTLP10* and *FuTLP25* to 25 in *FuTLP13*. CEs involved in plant development were also identified in the promoters of *FuTLP* genes (Figure 7, Table S7). Among the 172 elements discovered, the most numerous were the AAGAA-motif, CAT-box, and O2-site. The highest number of CEs putatively involved in developmental processes was observed in *FuTLP22* (11 CEs), while *FuTLP1*, *FuTLP9*, and *FuTLP13* had only two such elements.

**Figure 8:**
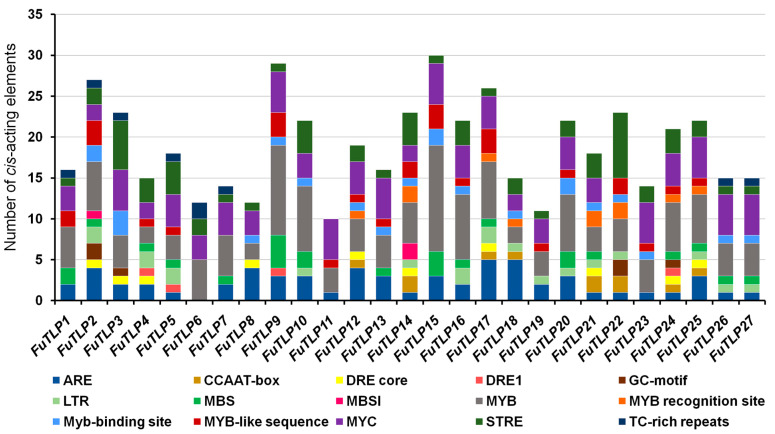
Distribution of *cis*-acting regulatory elements responsive to abiotic stress in promoters of *FuTLP* genes.

The group of abiotic stress-responsive elements included 510 CEs (Figure 7 and Figure 8). The highest number (30 CEs) of these elements was found in *FuTLP15*, and the lowest number (10 CEs) was discovered in *FuTLP11*. The most numerous CEs, MYB and MYC, putatively involved in responses to abiotic stress and in plant developmental processes, were discovered in all genes (Figure 8, Table S7) [66,67]. A stress-responsive element (STRE) that initiates transcriptional regulation of genes in response to diverse stresses was found in all *FuTLP* genes except *FuTLP22*. An anaerobic-responsive element (ARE) essential for anaerobic induction was found in all *FuTLP* genes except *FuTLP6* (Figure 8, Table S7). It is noteworthy that a low-temperature responsive element (LTR) was detected in 15 *FuTLP* genes: *FuTLP2*, *4*, *5*, *10*, *14*, *16*, *17*, *18*, *19*, *20*, *21*, *22*, *25*, *26*, and *27*. Therefore, the expression of *FuTLP* genes may be regulated by abiotic stress-responsive CEs.

**Figure 9:**
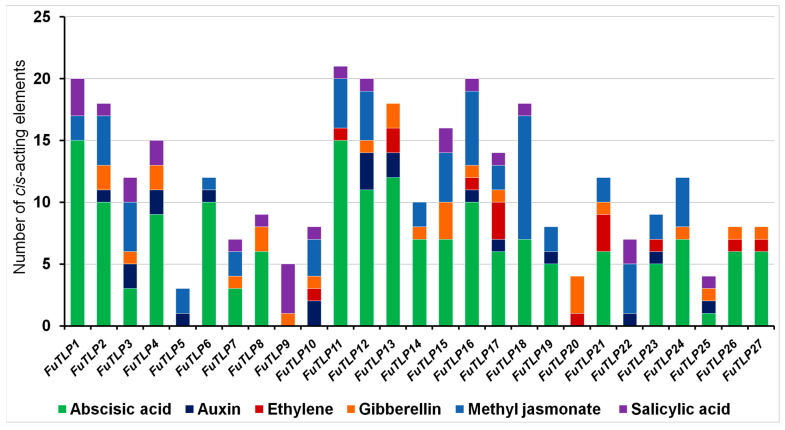
Distribution of *cis*-acting regulatory elements responsive to hormones in promoters of *FuTLP* genes.

The next group of CEs, including 318 sites that were found in the promoters of *FuTLP* genes, comprises elements responsive to hormones (Figure 9, Table S7). All *FuTLP*s contained hormone-responsive elements, with the majority putatively responding to at least two of the six hormones (Figure 9). Most *FuTLP*s (22) had CEs responsive to abscisic acid (ABA): ABRE, ABRE3a, ABRE4, and AT~ABRE. Less common were elements responsive to methyl jasmonate (CGTCA-motif, GTGGC-motif, TGACG-motif) and gibberellin (GARE-motif, P-box, TATC-box). Such CEs, as AuxRR-core, TGA-box, and TGA-element, are involved in auxin responsiveness. TCA and TCA-elements participate in the response to salicylic acid. The ERE element is sensitive to ethylene.

**Figure 10:**
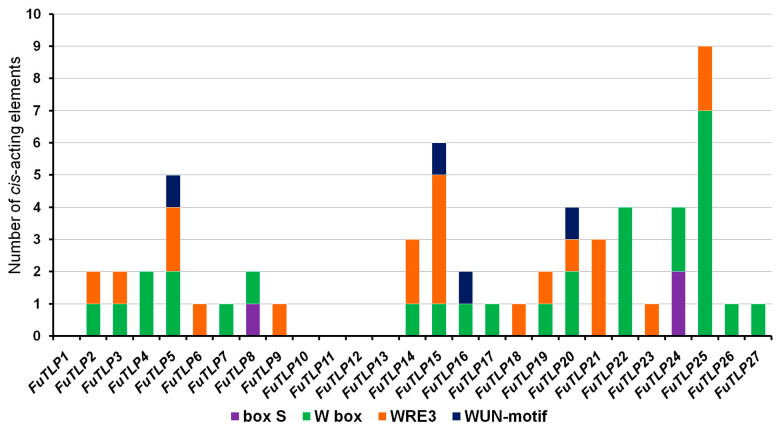
Distribution of *cis*-acting regulatory elements responsive to biotic stress in promoters of *FuTLP* genes.

The smallest group, consisting of 58 CEs, includes those responsive to biotic stress (Figure 10, Table S7). *Cis*-elements, such as box S, W box, WRE3, and WUN-motif, interact with transcription factors under biotic stress conditions and act as damage-responsive elements [68]. The most numerous of these elements, the W-BOX, responds to wounding and fungal elicitors and was detected in *FuTLP2*, *3*, *4*, *5*, *7*, *8*, *14*, *15*, *16*, *17*, *19*, *20*, *22* (4 CEs), *24*, *25* (7 CEs), *26*, and *27*. It is also worth noting that *FuTLP1*, *10*, *11*, *12*, and *13* did not have any CEs involved in biotic stress responses.

## 4. Discussion

PR-5 family proteins were discovered in different plant species several decades ago. They are encoded by multigene families and found not only in plants, but in animals and fungi as well. Plant PR-5 proteins comprise polypeptides with considerable sequence similarity among family members both within and between species and a common fold of the molecule. The concept that PR-5 family proteins are involved in defense against biotic and abiotic stresses and in developmental processes has been well substantiated by experimental data [7]. Although considerable progress has been achieved in PR-5 protein family research, the biological activity of the individual members, the mode of action, and the determinants of antifungal activity remain largely unexplored and are only beginning to be clarified.

Meadowsweet *F. ulmaria* (2*n* = 14) is a well-known medicinal plant used in traditional medicine to cure various diseases. Beyond its ability to alleviate pain, meadowsweet exhibits anti-inflammatory, antibacterial, and antioxidant effects, which can be used to treat various conditions such as colds, flu-like illnesses, and joint or muscle pain. The role of PR proteins in the biological activities of meadowsweet has not been studied so far. In this work, we performed a genome-wide analysis of *F. ulmaria* TLP genes, belonging to the PR-5 protein family, studied the structure of *FuTLP* genes and encoded proteins, and examined transcription of *FuTLP* genes in response to the fungal infection.

In the meadowsweet genome, we identified 27 putative TLP genes named *FuTLP1–27*. In a closely related diploid species, *R. chinensis*, the same number of TLP genes was discovered by genome-wide analysis [36]. In contrast, in an octaploid cultivated strawberry, *Fragaria* × *ananassa,* belonging to the same Rosaceae family, 76 TLPs were identified [37]. Thus, the number of TLP genes varies in different species.

The *FuTLP* genes were unevenly distributed on all seven chromosomes. The highest number of genes (8) was found on chromosome 6, and only two genes were identified on chromosomes 1, 2, and 7 (Figure 1a). On chromosome 6, a cluster of six genes, including *FuTLP18*–*FuTLP23*, was discovered. Several *FuTLP* genes formed pairs of genes located in close proximity to each other: *FuTLP1* and *FuTLP2* on chromosome 1; *FuTLP6* and *FuTLP7* on chromosome 3; *FuTLP9* and *FuTLP10*, as well as *FuTLP12* and *FuTLP13* on chromosome 4; and *FuTLP26* and *FuTLP27* on chromosome 7. Clustering of multiple TLP genes on the same chromosome suggests tandem duplication as one of the possible mechanisms for the expansion of the TLP family in meadowsweet. As a driving force of evolution, duplications often lead to neofunctionalization—the acquisition of new functions—for the duplicated genes. We observed the diversification of functions among the TLP family members (see below).

Most *FuTLP* genes contained one or two introns. Eight genes (*FuTLP4*, *13*, *18*, *19*, *20*, *21*, *22*, *23*) carried no introns (Figure 1b). Note that six of them were located in the gene cluster on chromosome 6. The transcription and translation of genes without introns occur more quickly than that of intron-containing genes due to the absence of splicing of primary transcripts, which is important for a quick response to environmental stressors.

*F. ulmaria* TLPs are synthesized as precursor proteins containing a signal peptide that mediates transport across the membrane of the endoplasmic reticulum and subsequent transport to other organelles or extracellular space [69]. Most FuTLPs are predicted to be secreted to the extracellular space, and only 6 (FuTLP18, 19, 20, 21, 22, 23) are localized in the vacuole. This finding suggests that these six FuTLPs are functionally different from the other FuTLPs.

Prediction of physicochemical properties of FuTLPs showed that the family comprises acidic, neutral, and basic proteins, which is typical for TLP families from other plants (Table 1 and Table 3). All FuTLPs contain a signature G-x-{GF}-x-C-x-T-{GA}-D-C-x(1,2)-{GQ}-x(2,3)-C, characteristic of the plant TLP family. They also have 16 cysteine residues found in “long” plant TLPs in conserved positions that form disulfide bonds. Two extra cysteine residues of FuTLP15 and an extra cysteine residue of FuTLP8, FuTLP27, and FuTLP11 are not involved in the formation of disulfide bonds and do not affect the spatial structure of the proteins. Furthermore, four TLPs, FuTLP1, 2, 10, and 14, have additional cysteines in the C-terminal extensions (Figure S1).

FuTLPs show sequence similarity of 57–91% to the TLPs from Rosaceae plants. Analysis of the conserved motifs in FuTLP sequences with MEME disclosed seven motifs arranged in the same order in most FuTLPs; however, seven proteins (FuTLP18, 19, 20, 21, 22, 23, 24) lacked one of the motifs, and in FuTLP14, it was located at the C-terminal region of the molecule. It is of interest that again FuTLP18, 19, 20, 21, 22, and 23 stay apart from other FuTLPs. Several FuTLPs (FuTLP1, 2, 9, 10, 12, 13, 14, 23) have C-terminal extensions after the last conserved motif. The role of the C-terminal prodomain remains obscure. In *Solanum nigrum* osmotins, it is supposed to target them to the vacuole [70]. However, not all osmotins harbor the C-terminal propeptide. For example, soybean osmotins GmOLPa and P21, as well as P21-like and GmOLPa-like isoforms, do not have C-terminal extensions [71]. In meadowsweet, six FuTLPs (FuTLP18–23) were predicted to be located in the vacuoles (Table 1). However, only FuTLP23 possesses a C-terminal propeptide of 18 amino acid residues, suggesting that vacuolar transport of FuTLPs may not be associated with the C-terminal prodomain.

Molecular modeling shows that FuTLPs adopt a typical structure of plant TLPs, consisting of three domains with a V-shaped cleft between domains I and II, which is supposed to bind ligands or receptors (Figure 3 and Figure S2). In most FuTLPs, the cleft is composed of five conserved residues REDDD (the negative charge −3), which are assumed to be important for antifungal activity [7]. The five conserved residues are found in all FuTLPs except FuTLP4, 5, 17, 19, and 24, in which substitutions at positions II, III, and IV are observed (Figure S2). In FuTLP19, D is replaced by E; however, the same negative charge of the cleft is retained; in FuTLP5, the acidic glutamic acid is replaced for neutral Q, just reducing the negative charge of the cleft by 1; in addition to this, in FuTLP4 there was another substitution of the first aspartic acid residue for G, which led to a reduced negative charge of −1; while in FuTLP24, three amino acid residues EDD are substituted for QSN, resulting in the neutral charge of the pentapeptide in the cleft. We may suggest that FuTLP24 exhibits other functions than the remaining meadowsweet TLPs; however, this suggestion requires experimental verification.

Phylogenetic analysis of *F. ulmaria* TLPs, in combination with *A. thaliana* TLPs and *R. chinensis* TLPs, showed that they fall into 10 clusters, with an uneven distribution of family members among the clusters (Figure 4). Groups V and VI were the most abundant. It is worth noting that group V includes TLP AT4G11650 (ATOSM34) from *Arabidopsis* involved in the defense response to pathogens and environmental stress, and RcTLP21 and RcTLP23 from rose, responsive to drought and salt stress [36,72,73,74], suggesting that *FuTLP18–23* in this cluster might have similar functions. It is also known that the TLP AT1G75800 from *Arabidopsis*, belonging to group VI, participates in defense responses against both biotic and abiotic stresses, and several rose TLPs from the same group (RcTLP6, RcTLP7, RcTLP8, RcTLP24, and RcTLP27) are involved in drought and salt response [36,75]. We may speculate that eleven meadowsweet TLPs in this Group VI have similar properties. However, this suggestion needs experimental validation.

Studies of the *R. chinensis* TLPs at three different stages of flower development showed that genes of six RcTLPs (RcTLP3 (Group VII), RcTLP4 (Group I), RcTLP6 (Group VI), RcTLP16 (Group VI), RcTLP20 (Group III), and RcTLP27 (Group VI)) have high expression levels in the ovary, tube, and flower [36]. It can be hypothesized that meadowsweet TLPs in these clusters may also play a role in flower development.

Transcriptomic analysis of *F. ulmaria* after infection with *B. sorokiniana* showed that of 27 TLP genes found in the genome, 12 were expressed under the experimental conditions used. Infection with the fungus led to considerable changes in the expression of six TLP genes. In *F. ulmaria*, 24 h after pathogen inoculation, three genes, *trFuTLP14*, *trFuTLP18*, and *trFuTLP21*, were down-regulated (Figure 5). After 48 h, three other genes, *trFuTLP20*, *trFuTLP22*, and *trFuTLP23*, were up-regulated while *trFuTLP14* was still down-regulated. It is of particular interest that the meadowsweet differentially expressed genes (with one exception, *trFuTLP14*) have no introns (see above), and thus can be rapidly expressed in response to infection or other types of stress. The up-regulated genes—*trFuTLP20*, *trFuTLP22*, and *trFuTLP23*—are, thus, involved in response to *B. sorokiniana* infection. Notably, only the genes from the phylogenetic Group V associated with stress response were up-regulated in *F. ulmaria*. Some meadowsweet TLP genes are not responsive to *B. sorokiniana* infection. This observation suggests that these constitutively expressed genes may participate in the physiological processes that are not associated with biotic stress.

Our results on up-regulation of meadowsweet TLP genes in response to infection are supported by studies on other plants. For example, in rose, following infection with the fungal pathogen *Botrytis cinerea*, up-regulation of three *RcTLP* genes (*RcTLP23*, *RcTLP6*, and *RcTLP7*) was observed, with the maximum expression level occurring within 48 h after infection [36]. In *Vitis vinifera*, high expression levels of osmotin and other thaumatin-like protein genes were observed in leaves and berries after infection with *Uncinula necator*, *Phomopsis viticola*, and *B. cinerea* [76]. High expression level of thaumatin and osmotin genes in a resistant grapevine variety (*V. vinifera*, Chardonnay) correlated with suppression of growth of spores and hyphae of *Elsinoe ampelina* [77]. A thaumatin protein gene, *TaPR5* from wheat, was induced by stripe rust fungus [78]. Several TLP genes of *Fragaria* × *ananassa* were up-regulated after *Colletotrichum gloeosporioides* infection [37]. The TLP genes from garlic were differentially expressed in resistant and susceptible garlic cultivars in response to *Fusarium proliferatum* infection [31]. All these results support the idea that TLPs from different plants participate in defense responses against pathogens.

All promoters of *FuTLP* genes contain *cis*-acting regulatory elements associated with the activation of defense mechanisms in response to biotic and abiotic stress (such as anaerobic conditions, drought (dehydration), low temperature, and salinity) (Figure 7, Figure 8 and Figure 10). Additionally, CEs involved in hormone activation, responsible for initiating signaling pathways, were identified (Figure 9). A large number of CEs involved in the regulation of plant development throughout the entire vegetative period were also found. The presence of particular CEs in the promoters of *FuTLP* genes might point to possible functions of the corresponding proteins. Thus, TLP genes activated in response to *B. sorokiniana* infection—*trFuTLP20*, *trFuTLP22*, and *trFuTLP23*—are enriched in CEs associated with biotic stress response, supporting this suggestion. Nevertheless, the promoters of each *FuTLP* gene contain a variety of different CEs. Only a few *FuTLP*s, *FuTLP1*, *10*, *11*, *12,* and *13*, do not possess CEs related to activation of biotic stress response.

The abundance of regulatory elements in the promoter regions of TLP genes was reported for other plant species [29,30,31,35,36,37,38]. The presence of such a wide variety of regulatory elements in the promoters of TLP genes suggests that these genes are expressed under variable conditions and that they perform multiple functions in plants.

## 5. Conclusions

In summary, a total of 27 TLP genes were discovered in the *F. ulmaria* genome. The *FuTLP* genes are unevenly distributed across seven chromosomes, with chromosome 6 containing the highest number (8 genes). Several *FuTLP* genes have no introns; the remaining genes have one or two introns. Studies of the *FuTLP* promoter *cis*-acting elements revealed a multiplicity of predicted regulatory elements putatively associated with hormone signaling and abiotic and biotic stress responses. Subcellular localization predictions showed that most FuTLPs are located in the extracellular space and only six are in vacuoles. Multiple sequence alignment demonstrated that most TLPs possessed five conserved REDDD amino acid sequences associated with antifungal activity. Phylogenetic analysis with *A. thaliana* and *R. chinensis* TLPs grouped the FuTLPs into 10 clusters. Transcription profiling of meadowsweet TLP genes in response to *B. sorokiniana* infection revealed six differentially expressed genes, of which three genes, *trFuTLP20*, *22,* and *23*, were up-regulated, suggesting their involvement in defense response to fungal infection. This suggestion is supported by the intronless structure of the up-regulated genes, the presence of biotic stress-responsive *cis*-regulatory elements in their promoter regions, and phylogenetic analysis, grouping these genes together with TLPs from other plant species with established defensive roles. The combined data on the *F. ulmaria* TLP genes and proteins, phylogenetic analysis, *cis*-acting regulatory elements, and expression in response to stress indicate the divergence of functions among FuTLP family members. Overall, we comprehensively studied the TLPs genes and proteins in meadowsweet and their involvement in response to fungal infection. The results obtained offer valuable clues for understanding the various biological functions of FuTLPs. Future studies will disclose their specific role in resistance to biotic and abiotic stressors and developmental processes and elucidate the structural basis of their biological activity.

## Figures and Tables

**Table 1:** Characteristics of the predicted FuTLPs.

Name	Blast Homology	Accession Number	Identity, %	Molecular Weight, Da	Length, aa	Signal Peptide, aa	TMHs	Subcellular Localization	pI	Net Charge at pH 7	Aliphatic Index	GRAVY
FuTLP1	PREDICTED: thaumatin-like protein 1b [F.v.]	XP_004287804.1	80	31,838.45	312	22	1	Extracellular	4.46	−10	59.00	0.006
FuTLP2	thaumatin-like protein 1b [R.r.]	XP_062010792.1	83	31,118.01	304	22	0	Extracellular	4.34	−10	67.29	0.174
FuTLP3	thaumatin-like protein 1 isoform X2 [P.p.]	XP_020416932.1	80	25,965.71	244	24	0	Extracellular	8.14	3	58.65	−0.149
FuTLP4	osmotin-like protein [R.r.]	XP_062015632.1	88	26,942.83	251	21	0	Extracellular	8.48	5	66.75	−0.146
FuTLP5	PREDICTED: thaumatin-like protein [F.v.]	XP_004302370.1	84	28,481.48	259	23	0	Extracellular	7.52	1	63.61	−0.275
FuTLP6	hypothetical protein DVH24_000808 [M.d.]	RXI00574.1	82	25,693.86	245	26	1	Extracellular	4.24	−10	57.95	−0.145
FuTLP7	hypothetical protein DVH24_000808 [M.d.]	RXI00574.1	81	25,801.87	247	21	1	Extracellular	4.20	−10	55.71	−0.165
FuTLP8	thaumatin-like protein [R.r.]	XP_062002078.1	83	25,768.63	244	21	0	Extracellular	7.54	1	73.33	0.025
FuTLP9	thaumatin-like protein 1b [R.c.]	XP_024161880.1	91	29,434.79	276	22	0	Extracellular	8.67	7	68.8	−0.025
FuTLP10	PREDICTED: thaumatin-like protein 1b [F.v.]	XP_011457931.1	85	34,494.34	337	25	0	Extracellular	4.29	−14	52.13	−0.095
FuTLP11	PREDICTED: pathogenesis-related protein 5 [F.v.]	XP_004293225.2	83	26,275.51	256	22	1	Extracellular	4.47	−11	57.39	0.018
FuTLP12	pathogenesis-related thaumatin-like protein 3.5 [A.a.]	XP_050367124.1	82	33,631.54	321	25	1	Extracellular	4.69	−12	54.55	−0.218
FuTLP13	thaumatin-like protein 1 [R.s.]	KAM5565145.1	75	32,763.63	320	24	1	Extracellular	4.42	−9	56.66	−0.043
FuTLP14	hypothetical protein M0R45_011253 [R.a.]	KAK9945754.1	84	30,560.88	285	27	0	Extracellular	7.50	1	69.46	−0.037
FuTLP15	thaumatin-like protein [P.y.]	PQQ06625.1	85	26,150.37	246	28	0	Extracellular	8.52	6	78.44	0.062
FuTLP16	thaumatin-like protein 1 [P.p.]	XP_007222615.1	64	27,126.49	256	21	0	Extracellular	4.5	−9	54.89	−0.248
FuTLP17	thaumatin-like protein 1 [P.p.]	XP_007222615.1	65	26,869.28	254	25	2	Extracellular	5.09	−4	56.87	−0.171
FuTLP18	thaumatin-like protein 1 [R.r.]	XP_062029155.1	83	24,575.49	227	24	0	Vacuole	4.73	−4	41.74	−0.423
FuTLP19	thaumatin-like protein 1 [R.r.]	XP_062029155.1	78	24,777.98	228	26	0	Vacuole	5.4	−3	49.26	−0.440
FuTLP20	protein P21 [R.c.]	XP_024173800.1	89	24,702.62	229	26	0	Vacuole	5.27	−1	45.67	−0.443
FuTLP21	thaumatin-like protein 1 [R.r.]	XP_062029155.1	84	24,603.55	227	28	1	Vacuole	4.87	−3	42.24	−0.420
FuTLP22	protein P21 [R.c.]	XP_024173800.1	86	24,940.1	229	26	1	Vacuole	8.35	4	47.61	−0.464
FuTLP23	thaumatin-like protein 1 [R.c.]	XP_024173799.1	85	25,903.14	238	28	0	Vacuole	7.92	2	45.89	−0.463
FuTLP24	hypothetical protein M0R45_024472 [R.a.]	KAK9927280.1	57	23,222.44	212	19	0	Extracellular	9.18	11	54.66	−0.376
FuTLP25	thaumatin-like protein [P.d.]	XP_034216898.1	86	26,464.36	250	21	0	Extracellular	8.86	9	62.88	−0.148
FuTLP26	thaumatin-like protein 1b [R.c.]	XP_024176062.1	79	26,072.52	243	28	0	Extracellular	4.91	−5	52.75	−0.255
FuTLP27	thaumatin-like protein 1 [R.r.]	XP_061991270.1	85	25,884.07	249	21	0	Extracellular	4.4	−8	58.96	−0.031

The following abbreviations were used: *Rubus argutu*s, R.a.; *Rosa rugosa*, R.r.; *Rosa chinensis*, R.c.; *Rosa sericea*, R.s.; *Prunus dulcis*, P.d.; *Fragaria vesca*, F.v.; *Prunus persica*, P.p.; *Argentina anserina*, A.a.; *Malus domestica*, M.d.; *Prunus yedoensis* var. *nudiflora*, P.y.

**Table 2:** Results of the comparison of nucleotide sequences of meadowsweet TLP genes found in transcriptome (*trFuTLPs*) and genome (*FuTLPs*), as well as amino acid sequences of the encoded proteins.

Gene Name		Nucleotide Sequence Identity, % (bp) *	Amino Acid Sequence Identity, % (aa) **
*trFuTLP*	*FuTLP*		
*trFuTLP2*	*FuTLP2*	99.89 (1)	100.00 (0)
*trFuTLP4*	*FuTLP4*	99.49 (4)	99.63 (1)
*trFuTLP8*	*FuTLP8*	99.61 (3)	99.62 (1)
*trFuTLP10*	*FuTLP10*	99.72 (3)	99.71 (1)
*trFuTLP11*	*FuTLP11*	99.88 (1)	99.64 (1)
*trFuTLP13*	*FuTLP13*	99.90 (1)	99.69 (1)
*trFuTLP14*	*FuTLP14*	100.00 (0)	100.00 (0)
*trFuTLP18*	*FuTLP18*	98.48 (11)	97.19 (7)
*trFuTLP20*	*FuTLP20*	95.62 (32)	95.22 (12)
*trFuTLP21*	*FuTLP21*	100.00 (0)	100.00 (0)
*trFuTLP22*	*FuTLP22*	97.26 (20)	96.02 (10)
*trFuTLP23*	*FuTLP23*	100.00 (0)	100.00 (0)

The number of nucleotide (*) and amino acid substitutions (**) is shown in parentheses.

**Table 3:** Characteristics of the predicted trFuTLPs.

Name	Blast Homology	Accession Number	Identity, %	Molecular Weight, Da	Length, aa	Signal Peptide, aa	TMHs	Subcellular Localization	pI	Net Charge at pH 7	Aliphatic Index	GRAVY
trFuTLP2	thaumatin-like protein 1b [R.r.]	XP_062010792.1	83	31,118.01	304	24	0	Extracellular	4.34	−10	67.29	0.174
trFuTLP4	osmotin-like protein [R.r.]	XP_062015632.1	88	26,952.87	251	23	0	Extracellular	8.48	5	66.75	−0.146
trFuTLP8	thaumatin-like protein [R.r.]	XP_062002078.1	95	25,767.65	244	22	0	Extracellular	7.89	2	73.00	0.023
trFuTLP10	PREDICTED: thaumatin-like protein 1b [F.v.]	XP_011457931.1	86	34,528.36	337	22	0	Extracellular	4.29	−14	50.89	−0.1
trFuTLP11	PREDICTED: pathogenesis-related protein 5 [F.v.]	XP_004293225.2	83	26,261.48	256	30	1	Extracellular	4.47	−11	58.66	0.046
trFuTLP13	thaumatin-like protein 1 [R.s.]	KAM5565145.1	75	32,797.65	320	27	1	Extracellular	4.42	−9	55.32	−0.046
trFuTLP14	hypothetical protein M0R45_011253 [R.a.]	KAK9945754.1	84	30,560.88	285	28	0	Extracellular	7.50	1	69.46	−0.037
trFuTLP18	thaumatin-like protein 1 [R.r.]	XP_062029155.1	83	24,623.53	227	26	0	Vacuole	4.92	−3	41.29	−0.443
trFuTLP20	protein P21 [R.c.]	XP_024173800.1	90	24,710.68	229	28	0	Vacuole	5.27	−1	46.62	−0.441
trFuTLP21	thaumatin-like protein 1 [R.r.]	XP_062029155.1	84	24,603.55	227	26	1	Vacuole	4.87	−3	42.24	−0.420
trFuTLP22	protein P21 [R.c.]	XP_024173800.1	88	24,861.00	229	28	1	Vacuole	8.17	3	49.55	−0.406
trFuTLP23	thaumatin-like protein 1 [R.c.]	XP_024173799.1	85	25,903.14	238	19	0	Vacuole	7.92	2	45.89	−0.463

The following abbreviations were used: *Rubus argutus*, R.a.; *Rosa rugosa*, R.r.; *Rosa chinensis*, R.c.; *Rosa sericea*, R.s.; *Fragaria vesca*, F.v.

## Data Availability

The original contributions presented in this study are included in the article. Further inquiries can be directed to the corresponding author.

## Supplementary Materials

The following supporting information can be downloaded at https://www.mdpi.com/article/10.3390/cimb48060640/s1.

## Abbreviations

The following abbreviations are used in this manuscript:

PR proteins	Pathogenesis-related proteins
PR-5	Pathogenesis-related protein family 5
TLP	Thaumatin-like protein
AMPs	Antimicrobial peptides
pI	Isoelectric point
GRAVY	Grand Average of Hydropathy
CE	*Cis*-acting element
