# Supplementary material for: Genome-Wide Identification and Expression Analysis of the Thaumatin-like Protein Genes in Filipendula ulmaria under Bipolaris sorokiniana Infection
Source: Curr Issues Mol Biol. 2026 Jun 20;48(6):640. doi: 10.3390/cimb48060640 (PMC13298582; doi:10.3390/cimb48060640)
Supplement: Supplementary file 1 [file cimb-48-00640-s001.zip › Figure S1.pdf]

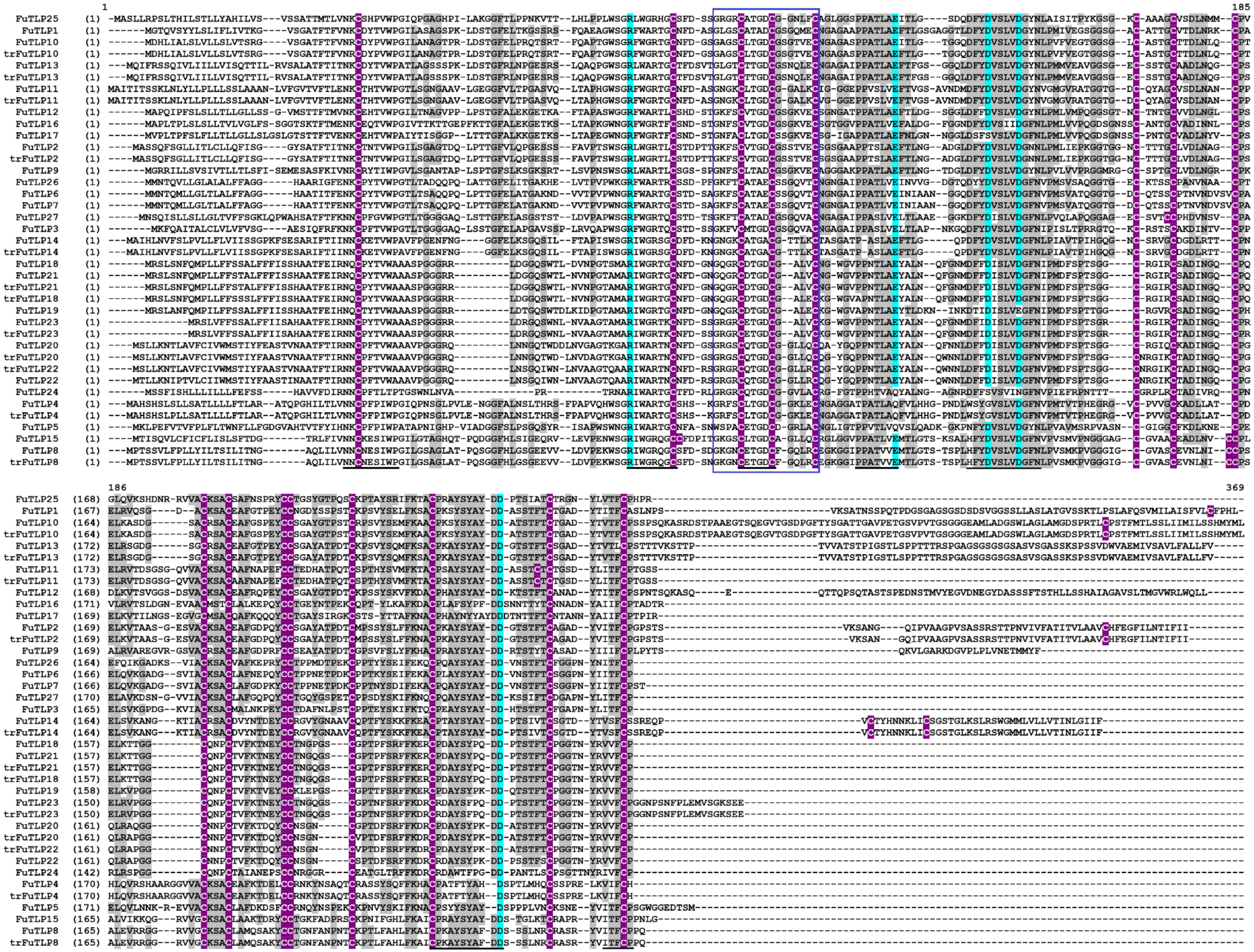

Figure S1. Multiple sequence alignment of meadowsweet TLP precursors. Five conserved residues REDDD are shaded cyan. Cysteine residues are shaded purple, and identical amino acids are shaded gray. The thaumatin-like motif is framed. Black lines below the sequences indicate seven conserved domains.
