# Supplementary material for: Genome-Wide Identification and Expression Analysis of the Thaumatin-like Protein Genes in Filipendula ulmaria under Bipolaris sorokiniana Infection
Source: Curr Issues Mol Biol. 2026 Jun 20;48(6):640. doi: 10.3390/cimb48060640 (PMC13298582; doi:10.3390/cimb48060640)

Figure S2. Predicted 3D structures of FuTLPs—surface representation. Non-polar residues are colored white, positively charged residues are colored blue, negatively charged residues are colored red, and negatively charged amino acids of REDDD motif located in the cleft are colored yellow.

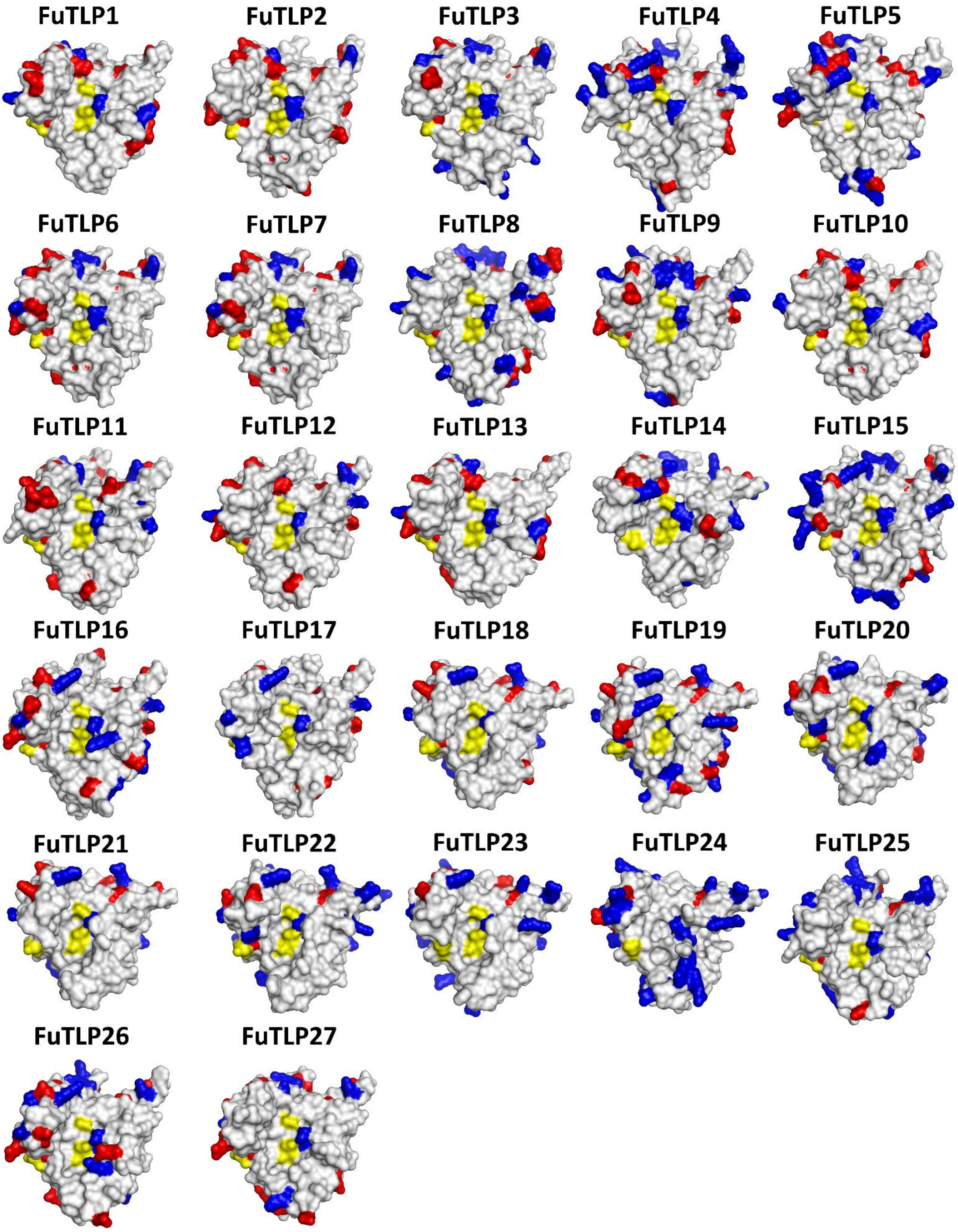

Supplement: Supplementary file 1 [file cimb-48-00640-s001.zip › Figure S2.pdf]
