# Supplementary material for: Genome-Wide Identification and Expression Analysis of the Thaumatin-like Protein Genes in Filipendula ulmaria under Bipolaris sorokiniana Infection
Source: Curr Issues Mol Biol. 2026 Jun 20;48(6):640. doi: 10.3390/cimb48060640 (PMC13298582; doi:10.3390/cimb48060640)
Supplement: Supplementary file 1 [file cimb-48-00640-s001.zip › Table S1.pdf]

Table S1. List of primers for validation of *trFuTLP* sequences by qRT-PCR.

№	Gene	Direct primer, 5'—3'	Reverse primer, 5'—3'	Length (bp)	T primer annealing (℃)
1.	*trFuTLP2*	ATAGGCAATGGCTTCCAGTC	TGCATTGGCATGCATCGTCG	1066	60
2.	*trFuTLP4*	CGTACCGACGTGAATCTACAG	CGGAAACTCACACAAGCTTAG	1003	60
3.	*trFuTLP8*	ATCAATCTCTGAAAACAAATGC	CAAGTTAAGGAGCTAGAACAT	867	60
4.	*trFuTLP10*	CCTGGTTTGCATTGTATGTA	CTCTCTGAAGAATTCACATTG	1113	60
5.	*trFuTLP11*	ATGCCATTCCCATCTACACTA	CACGTACTACATATGATGAC	949	60
6.	*trFuTLP13*	GATCAGTTGTTCTATAAGTTGG	CGACGATCTAGGCTGCATGTG	1126	60
7.	*trFuTLP14*	GAACATTCATAGACTTGCAG	CAACCAACATGGACAGTCTTG	1186	60
8.	*trFuTLP18*	AAACCCGTAATCTCAGTATC	ACTACTCTTGCATCGCGCGC	787	58
9.	*trFuTLP20*	CCAAGTCTATATAAAGCTCAT	AGCTTAACTTTAACACTTGG	814	60
10.	*trFuTLP21*	TCAGCAATCTACAAATCAAGC	ACTACTCTTGCATCGCGCGC	764	58
11.	*trFuTLP22*	CAAAATACACCAACTTAAACCC	CCTCGATCGTACATGCACTTAC	781	60
12.	*trFuTLP23*	CCAAGCTAGCTAGCGCTAATG	CTTGAAAGTAGACACCACAAC	872	60
