# Supplementary material for: Genome-Wide Identification and Expression Analysis of the Thaumatin-like Protein Genes in Filipendula ulmaria under Bipolaris sorokiniana Infection
Source: Curr Issues Mol Biol. 2026 Jun 20;48(6):640. doi: 10.3390/cimb48060640 (PMC13298582; doi:10.3390/cimb48060640)
Supplement: Supplementary file 1 [file cimb-48-00640-s001.zip › Table S2.pdf]

Table S2. List of primers for validation of *trFuTLP* expression levels by qRT-PCR.

№	Gene	Direct primer, 5'—3'	Reverse primer, 5'—3'	Length (bp)	T primer annealing (°C)
1.	*trFuTLP2*	GGCAGGACTCTATGCTCCAC	CCCTTGGGCTCGATCAACAT	212	60
2.	*trFuTLP4*	TTCAACGTCCCCATGACGG	GCGCTGTTGTACTTGTTCCTG	203	60
3.	*trFuTLP8*	TGGCCTGGAATTCTCGGAAG	TCCCCGGTTTCACAGTTTCC	173	60
4.	*trFuTLP10*	TTCAAGGCTGCTTGTCCCAA	AACCGGAACCGAACCAGTTT	243	60
5.	*trFuTLP11*	TATCCTCGTTAGCTGCTGCG	CCAGGGGTCAAGACGAAACC	145	60
6.	*trFuTLP13*	ATGCTTACGACGATGGGACG	GGCGACCTTGTTGTTGTTGG	163	60
7.	*trFuTLP14*	AGAGGGGTTTATGGGAACGC	GCTCCCTGGATGAGCAGAAT	163	60
8.	*trFuTLP18*	GCTAGTTAAATCAGAACCCAACCG	ATATGCGGGCCATGGAAGTC	299	58
9.	*trFuTLP20*	CCCACGTCCGGTGGGTGCAG	CAACTCTATAGTTAGTCCCAC	259	60
10.	*trFuTLP21*	TTTCAAGGAACGGTGTCCCG	TTCCCTCGTCATTTCAAGGGTT	321	58
11.	*trFuTLP22*	CCAACGTCCGGTGGGTGTAA	CAACTCTATAGTTAGTCCCAC	262	60
12.	*trFuTLP23*	GCATATGGGGCCGAACTAAG	ACCGAATTCCCCTGCACCGG	231	60
13.	*EF1-α*	TCATCATGAACCACCCTGGC	ACTTGGGCTCCTTCTCAAGC	144	58–60
