# Supplementary material for: Genome-Wide Identification and Expression Analysis of the Thaumatin-like Protein Genes in Filipendula ulmaria under Bipolaris sorokiniana Infection
Source: Curr Issues Mol Biol. 2026 Jun 20;48(6):640. doi: 10.3390/cimb48060640 (PMC13298582; doi:10.3390/cimb48060640)
Supplement: Supplementary file 1 [file cimb-48-00640-s001.zip › Table S3.pdf]

Table S3. Location of *FuTLP* genes on the chromosomes of meadowsweet and the number of exons in the genes.

Gene	Chromosome	Location on the chromosome	Number of exons
FuTLP1	1	OZ121597.1:33241871-33243176	3
FuTLP2	1	OZ121597.1:33251846-33253313	3
FuTLP3	2	OZ121596.1:9157675-9158551	2
FuTLP4	2	OZ121596.1:45025955-45026710	1
FuTLP5	3	OZ121598.1:12200326-12201297	2
FuTLP6	3	OZ121598.1:32391073-32391923	2
FuTLP7	3	OZ121598.1:32395103-32396079	2
FuTLP8	3	OZ121598.1:35434819-35435663	2
FuTLP9	4	OZ121599.1:8558154-8560090	3
FuTLP10	4	OZ121599.1:8580292-8581979	3
FuTLP11	4	OZ121599.1:21452624-21453495	2
FuTLP12	4	OZ121599.1:22982071-22983776	3
FuTLP13	4	OZ121599.1:22990526-22991488	1
FuTLP14	4	OZ121599.1:30654703-30655955	3
FuTLP15	5	OZ121600.1:11712153-11712999	2
FuTLP16	5	OZ121600.1:27754684-27755565	2
FuTLP17	5	OZ121600.1:29141692-29142558	2
FuTLP18	6	OZ121601.1:4360986-4361669	1
FuTLP19	6	OZ121601.1:4364870-4365556	1
FuTLP20	6	OZ121601.1:4375941-4376630	1
FuTLP21	6	OZ121601.1:4382839-4383522	1
FuTLP22	6	OZ121601.1:4386600-4387289	1
FuTLP23	6	OZ121601.1:4389134-4389850	1
FuTLP24	6	OZ121601.1:17192513-17193195	2
FuTLP25	6	OZ121601.1:21482856-21483743	2
FuTLP26	7	OZ121602.1:27550508-27551354	2
FuTLP27	7	OZ121602.1:28099058-28099951	2
