# Supplementary material for: Genome-Wide Identification and Expression Analysis of the Thaumatin-like Protein Genes in Filipendula ulmaria under Bipolaris sorokiniana Infection
Source: Curr Issues Mol Biol. 2026 Jun 20;48(6):640. doi: 10.3390/cimb48060640 (PMC13298582; doi:10.3390/cimb48060640)
Supplement: Supplementary file 1 [file cimb-48-00640-s001.zip › Table S6.pdf]

Table S6. Differential expression of *trFuTLP* genes in response to *B. sorokiniana* infection at 24 and 48 h post inoculation (hpi).

**24 hpi**			
**Gene**	**log2FoldChange**	**p-value**	**p-adjusted**
trFuTLP2	-0.52345	>0.05	>0.05
trFuTLP4	-0.31678	>0.05	>0.05
trFuTLP8	-0.21135	>0.05	>0.05
trFuTLP10	-0.73640	>0.05	>0.05
trFuTLP11	0.84313	>0.05	>0.05
trFuTLP13	-0.45091	0.00421	0.02834
trFuTLP14	-6.76766	0.00547	0.03469
trFuTLP18	-1.11475	0.00920	0.04186
trFuTLP20	0.07487	0.01480	>0.05
trFuTLP21	-1.44501	0.01984	>0.05
trFuTLP22	0.08403	>0.05	>0.05
trFuTLP23	0.67349	0.00920	0.04186
**48 hpi**			
**Gene**	**log2FoldChange**	**p-value**	**p-adjusted**
trFuTLP2	-0.61645	>0.05	>0.05
trFuTLP4	-0.40536	>0.05	>0.05
trFuTLP8	0.46404	>0.05	>0.05
trFuTLP10	-0.34501	>0.05	>0.05
trFuTLP11	0.31535	>0.05	>0.05
trFuTLP13	-0.07252	0.00421	0.02834
trFuTLP14	-2.59693	0.00468	0.08121
trFuTLP18	0.71761	0.00920	>0.05
trFuTLP20	1.60227	0.01480	>0.05
trFuTLP21	-0.46595	>0.05	>0.05
trFuTLP22	1.21391	0.03924	>0.05
trFuTLP23	1.11340	0.00193	0.01526
